# Pediatric humoral immune responses and infection risk after severe acute respiratory syndrome coronavirus 2 (SARS-CoV-2) infection and two-dose vaccination during SARS-CoV-2 omicron BA.5 and BN.1 variants predominance in South Korea

**DOI:** 10.3389/fimmu.2023.1306604

**Published:** 2023-12-20

**Authors:** Hyun-Woo Choi, Chiara Achangwa, Joonhong Park, Sun Min Lee, Nan Young Lee, Chae-Hyeon Jeon, Jeong-Hwa Choi, Hyun Kyung Do, Jeong-Hyun Nam, June-Woo Lee, Byoungguk Kim, Sukhyun Ryu, Seung-Jung Kee

**Affiliations:** ^1^ Department of Laboratory Medicine, Chonnam National University Hospital, Gwangju, Republic of Korea; ^2^ Department of Preventive Medicine, Konyang University College of Medicine, Daejeon, Republic of Korea; ^3^ Department of Laboratory Medicine, Jeonbuk National University Medical School and Hospital, Jeonju, Republic of Korea; ^4^ Department of Laboratory Medicine, Pusan National University School of Medicine, Pusan, Republic of Korea; ^5^ Department of Clinical Pathology, School of Medicine, Kyungpook National University, Daegu, Republic of Korea; ^6^ Chonnam National University Research Institute of Medical Science, BioMedical Sciences Graduate Program (BMSGP), Chonnam National University Medical School, Hwasun, Republic of Korea; ^7^ Division of Vaccine Clinical Research, Center for Vaccine Research, National Institute of Infectious Diseases, National Institute of Health, Korea Disease Control and Prevention Agency, Cheongju, Republic of Korea; ^8^ Department of Laboratory Medicine, Chonnam National University Medical School, Gwangju, Republic of Korea

**Keywords:** hybrid immunity, SARS-CoV-2, COVID-19, antibody response, child, vaccine

## Abstract

**Background:**

Humoral immune responses and infection risk after severe acute respiratory syndrome coronavirus 2 (SARS-CoV-2) infection and coronavirus disease 2019 (COVID-19) vaccination during the Omicron BA.5 and BN.1 variants predominant period remains unexplored in pediatric population.

**Methods:**

We examined anti-spike (anti-S) immunoglobulin G (IgG) responses in a total of 986 children aged 4−18 years who visited outpatient clinics between June 2022 and January 2023, with a history of SARS-CoV-2 infection alone, completed two doses of COVID-19 vaccination alone, vaccine-breakthrough infection (i.e., infection after the single dose of vaccination), and no antigenic exposure. Furthermore, to determine SARS-CoV-2 infection risk, the incidence of newly developed SARS-CoV-2 infection was investigated up to March 2023.

**Results:**

The anti-S IgG levels in the ‘vaccine-breakthrough infection’ group exceeded those in the ‘infection alone’ and ‘vaccination alone’ groups (both *P <*0.01). Furthermore, the ‘vaccination alone’ group experienced more rapid anti-S IgG waning than the ‘infection alone’ and ‘vaccine-breakthrough infection’ groups (both *P <*0.01). We could not identify newly developed SARS-CoV-2 infection in the ‘vaccine-breakthrough infection’ group.

**Conclusion:**

Our findings suggest that hybrid immunity, acquired from SARS-CoV-2 infection and COVID-19 vaccination, was a potentially higher and longer-lasting humoral immune response and protected against SARS-CoV-2 infection in pediatric population during Omicron BA.5 and BN.1 variants predominant.

## Introduction

Severe acute respiratory syndrome coronavirus 2 (SARS-CoV-2) is a highly transmissible virus that causes coronavirus disease 2019 (COVID-19) (1). South Korea, located in eastern Asia, has a population of 51.4 million, and it achieved significant success in mitigating the COVID-19 pandemic via public health and social measures alone without lockdown during the 2020–2021 period (2, 3). In January 2022, in South Korea, the SARS-CoV-2 Delta (B.1.617.2) variant was overtaken by the Omicron variant (B.1.1.529), which rapidly became the predominant variant (4). In March 2022, the daily incidence of COVID-19 cases reached approximately 600,000 owing to the Omicron variant’s aggressive nature, and this was the largest recorded increase in the number of new daily infections globally since the pandemic began (5).

In February 2021, in South Korea, the adenoviral vectored vaccine (ChAdOx1 nCoV-19) against SARS-CoV-2 was initially authorized for adults, and in March 2021, the mRNA vaccine (BNT162b2) was introduced for children aged >5 years (6). Immunity induced by natural SARS-CoV-2 infection and COVID-19 vaccination is known to reduce SARS-CoV-2 infection risk, hospitalization, and morbidity caused by SARS-CoV-2 (7–10). Furthermore, hybrid immunity acquired from both natural SARS-CoV-2 infection and COVID-19 vaccination reportedly generates greater humoral immunogenicity than either vaccination or natural SARS-CoV-2 infection alone (11). Although SARS-CoV-2 seroprevalence studies, including those focusing on immune response and COVID-19 vaccine effectiveness, have been conducted in various locations across several countries (11–13), most findings have largely been based on the adult population, and children have rarely been included.

Therefore, this study examined humoral immune responses in terms of the magnitude and durability of mRNA COVID-19 vaccination and/or previous SARS-CoV-2 infection in the child population. It also compared SARS-CoV-2 infection risk among children who were infected (unvaccinated, vaccinated, and/or had no antigenic exposure) to SARS-CoV-2 between June 2022 and March 2023, corresponding to the period in which the Omicron BA.5 and BN.1 variants were sequentially dominant.

## Methods

### Study design

A cross-sectional serosurvey was conducted between 16 June 2022 and 5 January 2023. Serum samples were conveniently collected from individuals aged 4–18 years who visited the outpatient clinics of four national regional pediatric hospitals located in Jeollanam, Jeollabuk, Gyeongsangsnam, and Gyeongsangsbuk provinces.

Children were invited to participate alongside an adult legal guardian. All guardians who had consented to participate were asked to respond to an in-person survey administered by trained staff and allow their children to provide blood specimens, which were collected at the respective pediatric hospitals. The exclusion criteria were as follows: (1) the presence of any respiratory symptom, (2) a history of COVID-19-related home quarantine, and/or (3) any contact history with a COVID-19 case within the preceding 14 days. Participant data included age, sex, comorbidities, history of previous SARS-CoV-2 infection, and COVID-19 vaccination status. Furthermore, with approval from the study participants, the specific, individual dates of previous SARS-CoV-2 infections and COVID-19 vaccinations were extracted from the national infectious disease notification and vaccine registry database of the Korea Disease Control and Prevention Agency to avoid recall bias. By that time, mRNA vaccines (BNT161b2) had been administered to pediatric population in South Korea, and each dose was recorded 14 days after administration. This study was approved by the institutional review boards of Chonnam National University Hospital (IRB No. CNUH-2022-118), Chonbuk National University Hospital (IRB No. CUH-2022-06-031-007), Kyungpook National University Chilgok Hospital (IRB No. KNUCH-2022-05-008-001), and Pusan National University Hospital (IRB No. PNUH-05-2022-103). Informed consent was obtained from all study participants (over 5-year-old) and all study participants’ guardians.

### Laboratory immunoassay

To detect anti-SARS-CoV-2 antibodies, two commercially available anti-SARS-CoV-2 immunoglobulin immunoassays were employed. The Elecsys^®^ Anti-SARS-CoV-2 N immunoassay (Roche Diagnostics International Ltd., Rotkreuz, Switzerland), with a sensitivity of 99.8% and specificity of 99.1% (14–16), was used to detect antibodies against the SARS-CoV-2 nucleocapsid protein (N), which are known to develop during a natural infection only (17). Therefore, anti-N IgG was measured to identify the previous SARS-CoV-2 infection in this study. Similarly, to measure antibodies against the SARS-CoV-2 spike protein (S), which are known to develop from both natural SARS-CoV-2 infection and COVID-19 vaccination (18), the Elecsys^®^ Anti-SARS-CoV-2 S immunoassay (Roche Diagnostics International Ltd., Rotkreuz, Switzerland), which detects the immunoglobulin specific for the S1 domain of the SARS-CoV-2 spike protein with a sensitivity of 99.6% and specificity of 99.8%, was used (15, 19). Both assays ran on the Cobas^®^ e801 analyzer (Roche Diagnostics International Ltd., Rotkreuz, Switzerland). The anti-N IgG immunoassay results were produced as a cut-off index (COI), where results ≥1.00 COI were considered positive. The anti-N and anti-S IgG measurement range were 1.00–125 COI, and 0.40–250 U/mL, respectively. Values lower than the limit of quantification was reported as <0.4 U/mL. Values above the upper limit were diluted up to 50-fold and reported as >12,500 U/mL. All tests were performed according to the manufacturer’s instructions.

### Temporal patterns of anti-SARS-CoV-2 IgG response with infection alone, vaccination alone, and vaccine breakthrough infection groups

This study’s primary outcome of interest was the SARS-CoV-2 anti-S IgG level observed over 365 days post-COVID-19 vaccination/SARS-CoV-2 infection (i.e., the date of the last vaccination or that of laboratory-confirmed infection, respectively). Serum anti-S IgG levels were compared among the following three groups: (1) ‘infection alone’ group (i.e., those who had a previous history of SARS-CoV-2 infection and did not receive COVID-19 vaccination), (2) ‘vaccination alone’ group (i.e., those who had no history of SARS-CoV-2 infection but received two doses of COVID-19 vaccination), and (3) ‘vaccine-breakthrough infection’ group (i.e., those who were infected with SARS-CoV-2 after a single dose of mRNA vaccine).

Considering the small number of participants who had received a single vaccine dose after infection (n=16) and a booster dose (i.e., third dose) of the COVID-19 vaccine (n=41), only those who had received the second vaccine dose (n=148) were considered.

To identify the different temporal kinetics among the three groups, we assessed the temporal trend of anti-S IgG values in samples. The smoothing spline was applied to the anti-S IgG values with the function of the last antigenic exposure date to visualize the temporal trends. Furthermore, to compare the anti-S IgG decay kinetics among the three groups, a regression model interaction term of vaccine/infection history and sampling time was conducted. The coefficient obtained from the model were used to estimate the waning rates (11).

### Adjusted comparisons of anti-S IgG response with infection alone, vaccination alone, vaccine-breakthrough infection groups

Adjusted and unadjusted comparisons of anti-S IgG among the three groups were conducted using univariable and multivariable linear regressions. These regressions considered possible predictors of the humoral immune response of anti-S IgG to SARS-CoV-2 infection and/or vaccination in the study population. To normalize data, anti-S IgG levels were converted to log_10_ values, and coefficients were subsequently exponentiated to enable result interpretation. The predictors included sex, age, comorbidities, and serum-specimen sampling time (i.e., time from the date of serum-specimen collection to that of last COVID-19 vaccination or laboratory-confirmed date of SARS-CoV-2 infection).

### Comparison of newly developed SARS-CoV-2 infection to infection alone, vaccination alone, and no antigenic exposure groups

To assess SARS-CoV-2 infection risk after previous infection and/or vaccination, newly confirmed COVID-19 cases among the study participants were evaluated up to 15 March 2023 using national infectious disease notification registry. Thereafter, logistic regression models were used to assess the association between predictor variables and SARS-CoV-2 infection risk. The covariates included in the models were: age, sex, region, comorbidity, antigenic exposure, and observation period (i.e., the time after sample collection by 15 March 2023). We defined the individuals who had no previous history of SARS-CoV-2 infection and COVID-19 vaccination with negative anti-S and anti-N IgG as a ‘no antigenic exposure group’. In the ‘antigenic exposure’ group, the ‘vaccine-breakthrough infection’ category did not record any new infections; hence, it was not included in the model. Crude and adjusted coefficients with 95% confidence intervals (CIs) were subsequently estimated.

Descriptive statistics for demographic characteristics were calculated with Fisher exact test. Mann-Whiteney U test and Kruskal Walis variance analysis were used to examine the differences of the data. A *P*-value < 0.05 was considered to indicate statistical significance in all analyses. All statistical analyses were performed in R (version 4.0.1 for Windows, R Foundation for Statistical, Computing, Vienna, Austria).

## Results

### Demographic and antigenic exposure characteristics of study participants

Between 16 June 2022 and 5 January 2023, 1,000 individuals were enrolled in this study, of whom 986 (99%) had complete serological and demographic data (**Supplementary Figure 1**). The median age of participants was 10 years (interquartile range: 8−13 years), 457 (46%) were male, and 476 (48%) had no comorbidities upon enrolment (**Table 1**).

**Table 1:** Clinical and demographic characteristics of study participants by SARS-CoV-2 and vaccination history.

Characteristics	SARS-CoV-2 infection alone (n=536)	Vaccination alone (n=73)			Vaccine after infection (n=5)	Vaccine breakthrough Infection ^a^ (n=127)	Unvaccinated and uninfected (n=245)	Total (n=986)
		1^st^ dose (n=2)	2^nd^ dose (n=52)	3^rd^ dose (n=19)				
Age, years								
Median (IQR)	9 (7−11)	9.5 (7.3−11.8)	14.0 (13.0−16.0)	15.0 (13.5−17.0)	7 (7−10)	14 (13−16)	9 (7−11)	10.0 (8.0−13.0)
Range (Min−Max)	14 (4−18)	9 (5−14)	12.0 (6.0−18.0)	6 (12.0−18.0)	6 (6−12)	9 (9−18)	4 (4−18)	14 (4.0−18.0)
Gender, n (%)								
Male	243 (45.3)	2 (100.0)	26 (50.0)	5 (26.3)	2 (40.0)	65 (51.6)	114 (46.5)	457 (46.3)
Female	293 (54.7)	0 (0.0)	26 (50.0)	14 (73.7)	3 (60.0)	61 (48.4)	131 (53.5)	529 (53.7)
Age group, years, n (%)								
4−6	87 (16.2)	1 (50.0)	3 (5.8)	0 (0.0)	0 (0.0)	0 (0)	46 (18.8)	137 (13.9)
7−12	407 (75.9)	0 (0.0)	8 (15.4)	3 (15.8)	4 (80.0)	12 (9.5)	166 (67.7)	601 (61.0)
13−15	29 (5.4)	1 (50.0)	26 (50)	8 (42.1)	1 (20.0)	78 (61.9)	25 (10.2)	168 (17.0)
16−18	13 (2.4)	0 (0.0)	15 (28.8)	8 (42.1)	0 (0.0)	36 (28.6)	8 (3.3)	80 (8.1)
Province, n (%)								
Jeollanam/ Gwangju	147 (27.4)	1 (50.0)	21 (40.4)	13 (68.4)	4 (80.0)	43 (34.1)	57 (23.3)	287 (29.1)
Jeollabuk/Jeonju	84 (15.7)	0 (0.0)	9 (17.3)	3 (15.8)	1 (20.0)	28 (22.2)	37 (15.1)	162 (16.4)
Gyeongsangbuk/ Daegu	130 (24.3)	0 (0.0)	15 (28.8)	2 (10.5)	0 (0.0)	29 (23.0)	62 (25.3)	238 (24.1)
Gyeongsangnam/ Busan	175 (32.6)	1 (50.0)	7 (13.5)	1 (5.3)	0 (0.0)	26 (20.6)	89 (36.3)	299 (30.3)
Time of collection, n (%)								
Jun 2022	14 (2.6)	0 (0.0)	2 (3.8)	1 (5.3)	1 (20.0)	3 (2.4)	8 (3.3)	29 (2.9)
Jul 2022	62 (11.6)	1 (50.0)	16 (30.8)	3 (15.8)	0 (0.0)	13 (10.3)	35 (14.3)	130 (13.1)
Aug 2022	85 (15.9)	0 (0.0)	6 (11.5)	2 (10.5)	0 (0.0)	18 (14.3)	43 (17.5)	154 (15.6)
Sept 2022	89 (16.6)	0 (0.0)	9 (17.3)	5 (26.3)	1 (20.0)	19 (15.1)	43 (17.5)	166 (16.8)
Oct 2022	69 (12.9)	0 (0.0)	3 (5.8)	3 (15.8)	0 (0.0)	14 (11.1)	39 (15.9)	129 (13.1)
Nov 2022	113 (21.1)	0 (0.0)	9 (17.3)	3 (15.8)	2 (40.0)	29 (23.0)	29 (11.8)	185 (18.8)
Dec 2022	90 (16.8)	1 (50.0)	6 (11.5)	2 (10.5)	1 (2.0)	22 (17.5)	45 (18.4)	167 (16.9)
Jan 2023	14 (2.4)	0 (0.0)	1 (1.9)	0 (0.0)	0 (0.0)	8 (6.3)	3 (1.2)	26 (2.6)
Comorbidities, n (%)								
None	279 (52.1)	1 (50.0)	19 (36.5)	6 (31.6)	0 (0.0)	50 (39.7)	120 (49.0)	476 (48.3)
Immune disorder	19 (3.5)	0 (0.0)	3 (5.8)	3 (15.8)	0 (0.0)	12 (9.5)	21 (8.6)	58 (5.9)
Others ^b^	257 (47.9)	1 (50.0)	33 (63.5)	13 (68.4)	5 (100.0)	73 (57.9)	117 (47.7)	501 (50.8)
Sampling time, median (IQR), days								
Time since infection	172.5 (123.0−222.0)							
Time since latest vaccination		475.5 (436.2−514.8)	223 (188.2−271.0)	175.0 (113.5−219.0)	46 (30.0−68.0)	272.5 (218.0−322.0)		

IQR, interquartile range; Max, maximum; n, number; Min, minimum; SARS-CoV-2, Severe Acute Respiratory Syndrome Coronavirus 2.

a Vaccine-breakthrough infected individuals are infected 14 days or more after the vaccination.

b Others: Cardiovascular, endocrine, respiratory, hepatorenal, malignant, neurological psychological diseases.

Overall, 205 (21%) study participants had received at least one dose of SARS-CoV-2 vaccination. Among the study participants, 16 (2%), 148 (15%), and 41 (4%) had received the first, second, and third vaccine doses, respectively (**Table 1**). In this study, study participants were categorized into three groups: the ‘infection alone’ (n=536), ‘vaccination alone’ (dose two only, n=52), and ‘vaccine-breakthrough infection’ (n=127) groups. The median sampling times for the three groups, assessed based on the latest antigenic exposure (i.e., SARS-CoV-2 infection or vaccination), were 173, 216, and 272 days for the SARS-CoV-2 ‘infection alone’, ‘vaccination alone’, and ‘vaccine-breakthrough infection’ groups, respectively (**Table 1**).

### Comparison of anti-SARS-CoV-2 IgG response to SARS-CoV-2 infection alone, vaccination alone, and vaccine-breakthrough infection groups

**Figure 1:**
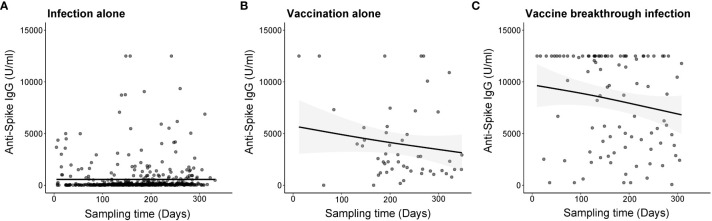
Temporal kinetics of anti-SARS-CoV-2 spike protein (anti-S) IgG by different types of antigenic exposures by time since SARS-CoV-2 infection or COVID-19 vaccination. Dots represent anti-S IgG values induced by **(A)** SARS-CoV-2 infection alone, **(B)** COVID-19 vaccination alone, and **(C)** vaccine breakthrough infection. The thick curve indicates the trend in anti-Spike IgG using smoothing splines with a 95% confidence interval (shaded area).

**Figure 2:**
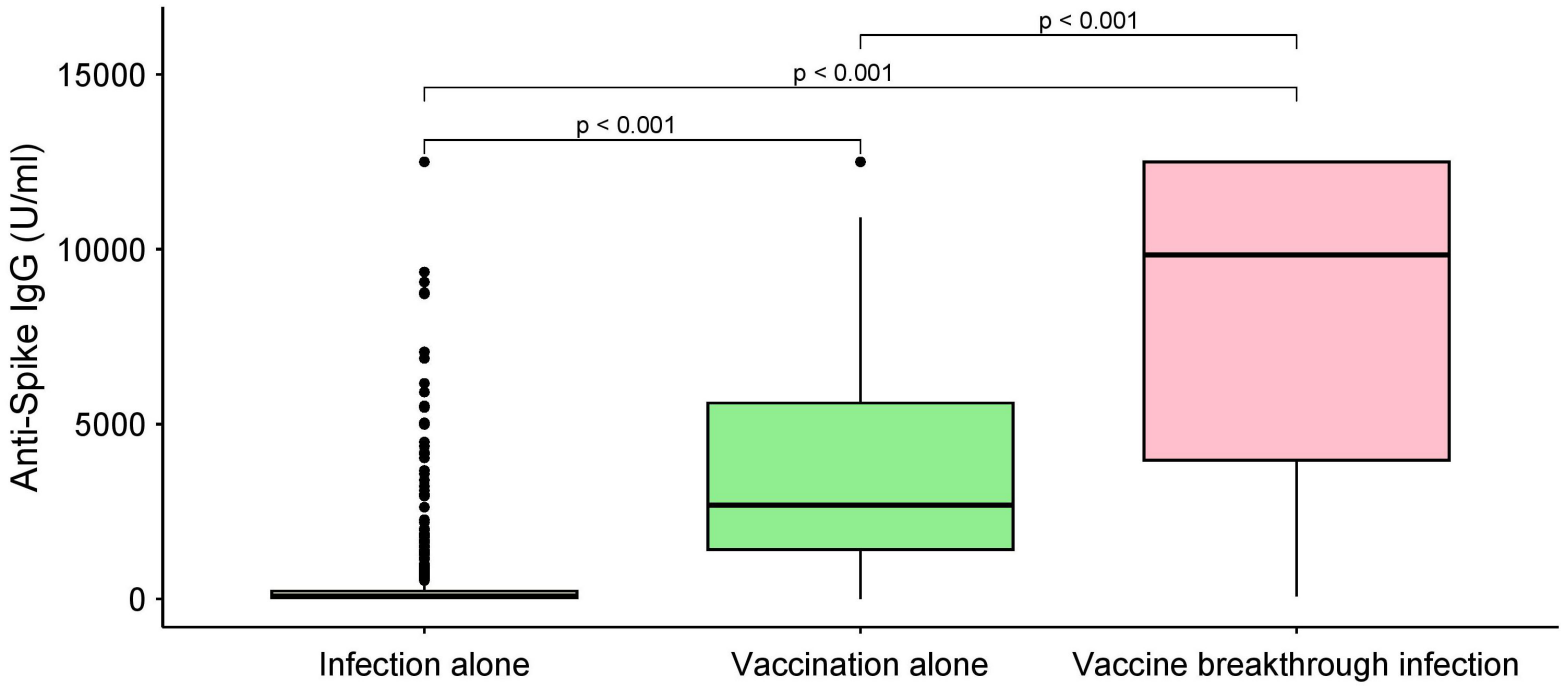
Unadjusted comparison of anti-S IgG by category of SARS-CoV-2 infection and/or COVID-19 vaccination. P-values determined using the Kruskal-Wallis test. The comparisons do not adjust for sampling time, which is different for every group. The box plots denote median, the 25th percentile, and the 75th percentile of the anti-S (y-axis) and each group (x-axis) representing SARS-CoV-2 infection alone (light yellow), vaccination alone (light green), and vaccine breakthrough infection (pink).

The kinetics of anti-S IgG by sampling time are presented in **Figure 1**. The median anti-S IgG levels were significantly higher in the ‘vaccine-breakthrough infection’ groups (median: 10,993, interquartile range (IQR): 4,331–12,500) than in the ‘infection alone’ (median, 73; IQR: 28–217) and ‘vaccination alone’ groups (median: 2678, IQR: 1,412–4,173) (both *P <*0.001, **Figure 2**). Similar results were observed after age stratification (**Supplementary Figure 2**).

On the regression model interaction term of vaccine/infection history and sampling time, compared with ‘infection alone group’, we identified the ‘vaccination alone’ group and the ‘vaccine-breakthrough infection’ group was associated with more rapid decay kinetics [coefficient (95% CI): 1.63 (0.08–34.12), and 1.27 (0.56–2.97), respectively] (**Table 2**).

**Table 2:** Longitudinal linear mixed modelling to estimate decay kinetics of log_10_ anti-S IgG response to antigenic exposure .

Covariate	Coefficient ^a^	*P*-value
Sampling time ^b^	1.001 (1.000−1.003)	<0.01
Vaccination alone	1.632 (0.081− 34.124)	0.002
Vaccine-breakthrough infection	1.271 (0.560 – 2.974)	<0.001
Sampling time × Vaccination alone ^c^	1.000 (0.997 – 1.001)	<0.001
Sampling time × Vaccine-breakthrough infection ^c^	0.993 (0.986−1.013)	<0.01

a Reference: Infection alone group.

b Sampling time refers to the last antigenic exposure (either time since the final vaccine was received or infection, whatever is latest).

c The × indicates interactions among the variables.

On our regression model after adjusting for demographic factors, including age, sex, region, comorbidities, and sampling time, ‘vaccination alone’ and ‘vaccine-breakthrough infection’ groups were associated with 3.43 (95% CI: 2.86**−**4.01, *P <*0.001) and 3.63 (95% CI: 3.13**−**4.34, *P* 0.002) increases in anti-S IgG levels compared with the ‘SARS-CoV-2 infection alone’ group (**Table 3**).

**Table 3:** Association between anti-S IgG, and demographic factors, type of antigenic exposure and sampling time in child population, South Korea.

Covariate	Crude coefficient (95% CI)	*P*-value	Adjusted coefficient ^a^ (95% CI)	*P*-value
Age group, years				
4−6	Reference		Reference	
7−12	0.12 (-0.42 to 0.67)	0.66	-0.28 (-0.66 to 0.01)	0.15
13−15	3.03 (2.38 to 3.70)	<0.001	-0.39 (-0.95 to 0.16)	0.16
16−18	3.83 (3.02 to 4.64)	<0.001	-0.33 (-0.95 to 0.29)	0.29
Gender				
Male	Reference		Reference	
Female	0.22 (-0.19 to 0.62)	0.296	0.15 (-0.12 to 0.37)	0.23
Province				
Jeollanam/Gwangju	Reference		Reference	
Jeollabuk/Jeonju	-0.32 (-0.94 to 0.35)	0.31	0.04 (-0.35 to 0.40)	0.82
Gyeongsangbuk/Daegu	-0.65 (-1.20 to -0.09)	0.02	0.10(-0.27 to 0.49)	0.59
Gyeongsangnam/Busan	-0.79 (-1.32 to -0.29)	0.003	0.05 (-0.36 to 0.57)	0.81
Comorbidity				
None	Reference		Reference	
Immune disorder	-0.66 (-1.69 to 0.35)	0.21	-0.34 (-0.66 to 0.64)	0.37
Others ^b^	0.01 (-0.31 to 0.51)	0.63	-0.32 (-0.69 to 0.07)	0.08
Antigenic exposure				
Infection alone	Reference		Reference	
Vaccine alone	3.36 (2.95 to 3.77)	<0.001	3.46 (2.86 to 4.01)	<0.001
Vaccine-breakthrough infection	4.07 (3.75 to 4.40)	<0.001	3.74 (3.13 to 4.34)	<0.001
Sampling time ^c^	0.01 (0.01 to 0.02)	< 0.001	0.002 (0.001 to 0.004)	0.01

CI, confidence interval.

a Adjusted for age group, sex, region, comorbidity, group, and sampling time.

b Others: Cardiovascular, endocrine, respiratory, hepatorenal, malignant, neurological psychological diseases.

c Sampling time refers to last antigenic exposure (time since final dose of vaccine or infection, whatever is latest).

### Comparison of newly developed SARS-CoV-2 infection to infection alone, vaccination alone, and no antigenic exposure groups

Overall, 78 study participants were identified newly confirmed COVID-19 cases after the SARS-CoV-2 infection and vaccination (**Supplementary Figure 3**). The median observation period, between the date of sampling collection and 25 March 2022 (i.e., the date of follow-up investigation for newly developed COVID-19), was 184.5 days (IQR: 144.0–224.0) (**Table 4**). After adjusting the demographic factors and observation period, compared with the ‘infection alone’ group, we identified ‘no antigenic exposure’ group and the ‘vaccination alone’ were associated with higher risk of developing COVID-19 [adjusted coefficient (95% CI): 2.32 (1.88–3.42), and 1.91 (1.61–3.16), respectively] (**Table 5**).

**Table 4:** Demographics of newly developed COVID-19 in child population during the observation period (time between sample collection and 25 May 2023) in South Korea.

Covariate	Participants (n=986)	Newly developed COVID-19 (n= 78)
Age group, years		
4–6	137	8 (5.8%)
7–12	601	50 (8.3%)
13–15	168	15 (8.9%)
16–18	80	5 (6.2%)
Sex		
Male	457	38 (8.3%)
Female	529	40 (7.6%)
Province		
Jeollanam/Gwangju	287	23 (8.0%)
Jeollabuk/Jeonju	162	13 (8.0%)
Gyeongsangbuk/Daegu	238	21 (8.8%)
Gyeongsangnam/Busan	299	21 (7.0%)
Comorbidities		
None	476	35 (7.3%)
Immune disorder	58	3 (5.2%)
Others^a^	501	40 (8.0%)
Observation period, months^b^		
3	38	1 (2.6%)
4	174	11 (6.3%)
5	166	9 (5.4%)
≥ 6	608	57 (9.4%)
Antigenic exposure		
Infection alone	536	60 (11.2%)
Vaccine alone	73	18 (24.6%)
None	158	37 (23.4%)

a Others: Cardiovascular, endocrine, respiratory, hepatorenal, malignant, neurological, and psychological diseases.

b Observation period refers to the time after sampling collection by 15 March 2023.

**Table 5:** Association between newly confirmed SARS-CoV-2 infection risk and covariates including demographic factors, type of antigenic exposure and sampling time in pediatric population, South Korea.

Covariate	Crude coefficient (95% CI)	*P*-value	Adjusted coefficient ^a^ (95% CI)	*P*-value
Age group, years				
4-6	Reference		Reference	
7-12	0.38 (-0.33 to 1.23)	0.33	0.50 (-0.06 to 1.36)	0.09
13-15	0.46 (-0.41 to 1.39)	0.31	0.30 (-0.73 to 1.26)	0.49
16-18	0.07 (-1.15 to 1.20)	0.90	-0.21 (-1.45 to 1.08)	0.72
Sex				
Male	Reference		Reference	
Female	-0.10 (-0.56 to 0.36)	0.66	-0.07 (-0.55 to 0.45)	0.85
Province				
Jeollanam/Gwangju	Reference		Reference	
Jeollabuk/Jeonju	0.001 (-0.73 to 0.69)	0.99	-0.16 (-0.92 to 0.60)	0.70
Gyeongsangbuk/Daegu	0.11 (-0.52 to 0.72)	0.74	-0.11 (-0.68 to 0.71)	0.91
Gyeongsangnam/Busan	-0.14 (-0.76 to 0.47)	0.65	-0.25 (-0.31 to 0.54)	0.61
Comorbidity				
None	Reference		Reference	
Immune disorder	-0.37 (-1.82 to 0.69)	0.54	-1.13 (-2.69 to 0.09)	0.09
Others ^b^	0.20 (-0.27 to 0.68)	0.40	-0.04 (-0.78 to 0.76)	0.77
Antigenic exposure				
Infection alone	Reference		Reference	
Vaccine alone	1.58 (1.13 to 2.92)	<0.001	1.91 (1.61 to 3.16)	<0.001
None	1.91 (1.42 to 3.01)	<0.001	2.32 (1.88 to 3.42)	<0.001
Sampling time ^c^				
Observation period, months ^d^				
3	Reference		Reference	
4	0.92 (-0.77 to 3.84)	0.39	1.04 (-0.70 to 3.99)	0.33
5	0.75 (-0.96 to 3.68)	0.48	0.86 (-0.90 to 3.81)	0.43
≥ 6	1.34 (-0.22 to 4.23)	0.19	1.43 (-0.18 to 4.34)	0.17

CI, confidence interval.

a Adjusted for age group, sex, region, comorbidity, antigenic exposure, sampling time, and observation period.

b Others: Cardiovascular, endocrine, respiratory, hepatorenal, malignant, neurological, and psychological diseases.

c Sampling time refers to last antigenic exposure (time since final dose of vaccine or infection, whatever is latest).

d Observation period refers to the time after sampling collection by 15 March 2023.

## Discussion

To the best of our knowledge, this is the first real-world study comparing the protective effectiveness in the pediatric population of immunity acquired from the two doses of COVID-19 vaccination and infection after the single dose of vaccination against SARS-CoV-2 infection during Omicron BA.5 and BN.1 variant predominant period.

Our analysis showed that more rapid decay IgG kinetics were observed for those in the vaccination-alone group compared to the infection-alone group. However, anti-S IgG titer in the population who received vaccination was larger than that of the SARS-CoV-2 infection alone group. This finding indicates that hybrid immunity acquired from both COVID-19 vaccination and SARS-CoV-2 infection potentially elicits greater humoral immunogenicity and longer durability than either two-dose COVID-19 vaccination alone or SARS-CoV-2 infection alone in the pediatric population.

Our findings also identified that children with two-dose mRNA vaccination did not exhibit statistically significant protection against SARS-CoV-2 infection during the Omicron BA.5 and BN.1 variant circulating period, compared with those with infection alone and no antigenic exposure group. This finding is in line with a previous study demonstrating that a single dose mRNA vaccination in the pediatric population did not confer significant protection against Omicron BA.4 and BA5 infection (20).

In this study, we could not identify newly developed SARS-CoV-2 infection in the ‘vaccine-breakthrough infection’ group. This is likely due to the higher humoral immune response after vaccination and infection than the vaccination-only or infection-only group (21, 22). Therefore, our finding provides insights into hybrid immunity, which affords a more robust protective effect against SARS-CoV-2 during Omicron BA.5 and BN.1 predominant period compared to immunity induced solely by infection and a two-dose vaccination. This interpretation is supported by previous studies that demonstrated the hybrid immune response provided superior protection against the Delta and Omicron (BA.1 and BA.2) variant than infection- or a single-dose vaccine-induced immunity alone (9, 21, 23–25). However, this also should be interpreted cautiously as COVID-19 vaccination reduces Omicron-associated hospitalization and severity of illness in the pediatric population (26).

**Figure 3:**
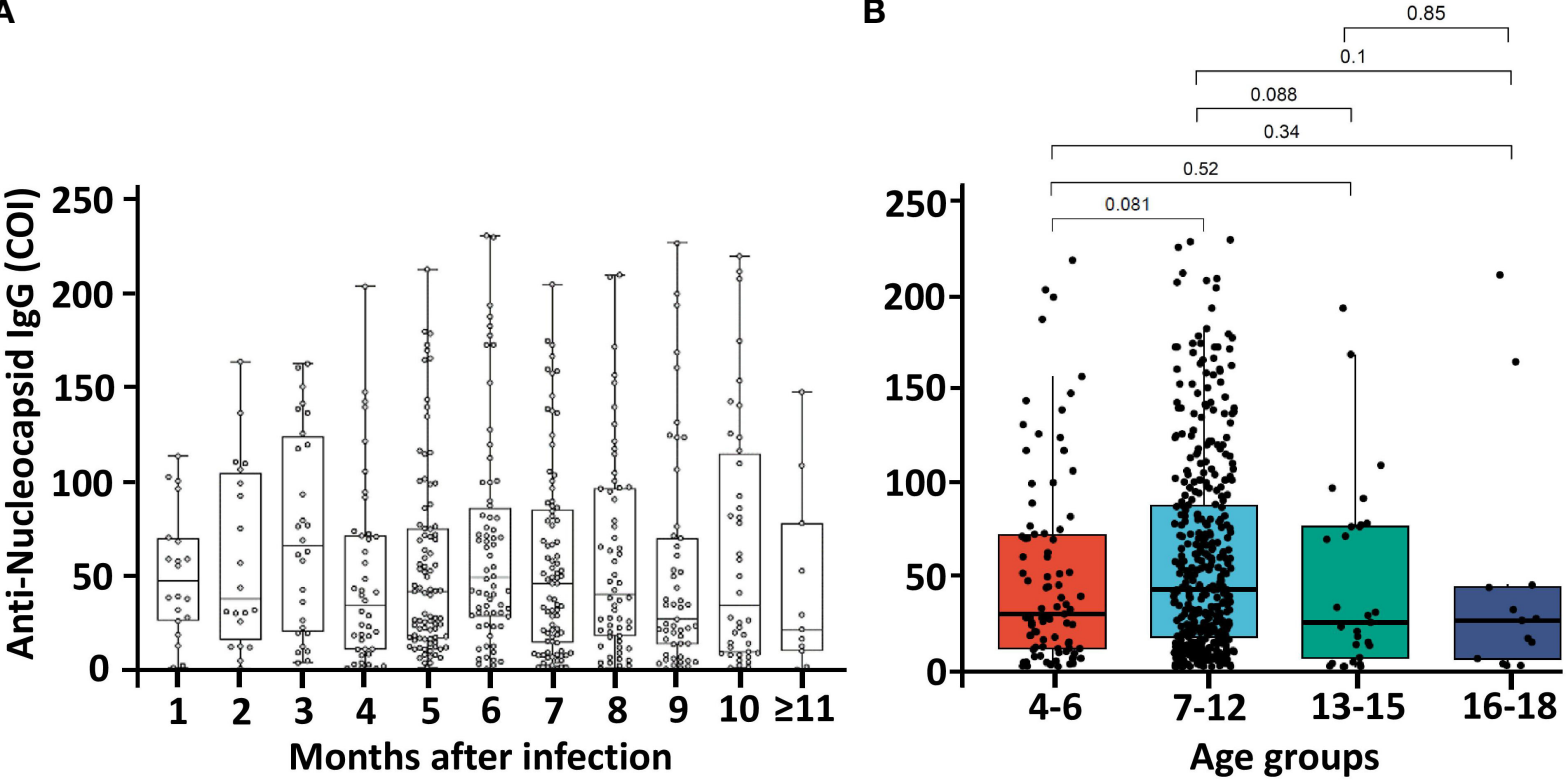
Temporal kinetics of anti-SARS-CoV-2 nucleocapsid (anti-N) IgG in SARS-CoV-2 infected groups (n=536) and comparison of anti-N IgG by different age groups. Dots represent an individual sample, and horizontal lines in the box indicate the median value with the interquartile range with **(A)** time in months from infection to collect samples and **(B)** different age groups. P- values were determined using the Kruskal-Wallis test. Abbreviation: COI, cut-off index.

We identified that 82% of the study population had an anti-S IgG response, which was positively associated with COVID-19 vaccination (27). This finding is in line with 89% of the child population had booster doses immunization in South Korea as of December 2022 (28). We also identified that 74% of this study’s population had an anti-N IgG response, which is known to be positively associated with previous SARS-CoV-2 infection (**Supplementary Figure 1**; **Supplementary Figure 3**). This indicates that a vast proportion of children had an immune response against natural SARS-CoV-2 infection. In this study, based on the anti-N seroprevalence and SARS-CoV-2 infection notification registry database in South Korea, 9.5% (95% CI: 7.8%−11.5%) of individuals did not report their SARS-CoV-2 infection to public health authorities. This finding is consistent with that of a previous report wherein 66% of the child population in South Korea was infected by SARS-CoV-2 between January 2020 and June 2022 (29). This finding needs to be interpreted cautiously owing to anti-N antibody waning, which reportedly persists for approximately 1 year after infection (30). However, in this study, we found that the temporal trend of the median anti-N IgG titer after SARS-CoV-2 infection exhibited a decreasing trend after 6 months but persisted for more than 11 months (**Figure 3A**), and a sizeable proportion of children in South Korea were infected after January 2022 during the Omicron wave. A previous study suggested that the decay rate of anti-N IgG was different in elderly (31). However, we could not identify any statistical difference in anti-N IgG distributions between the different age groups in our study (**Figure 3B**).

Previous studies have revealed differences between vaccination-induced and SARS-CoV-2-induced seroprevalence across varying demographics (13, 32). However, this study could not identify any significant sex- and age-related disparities in anti-S and anti-N IgG possibly because of the considerable number of children who had been infected by SARS-CoV-2 and had undergone COVID-19 vaccination in South Korea.

The key strength of this study is that it is the first pediatric population-based study that included a two-dose COVID-19 vaccination population and estimated infection risk during Omicron BA.5 and BN.1 predominant period. Furthermore, we used the South Korean national SARS-CoV-2 infection notification database, where the extended community COVID-19 screening center operated during the study period (4), and this is likely to provide improved estimates of the SARS-CoV-2 infection risk. However, it also has several limitations. First, the information of exact timing of SARS-CoV-2 infection is limited (33). Therefore, the reported anti-N and anti-S IgG titers could have been affected by the time of report. Second, the study population might have been biased because individuals who visited the medical facilities for sample collection were likely to be more health conscious. Furthermore, the study population might not represent the healthy pediatric population as the population was mainly recruited in outpatient clinics, and 52% had comorbidities. Therefore, a seroprevalence study using nationally representative samples of the general pediatric population may overcome this limitation. Third, we did not conduct neutralization assays to support our findings further. However, electrochemiluminescence immunoassay, which we conducted in this study, has strong positive correlation with neuralization assay (34, 35) and been widely used in serosurvey of large populations (11, 13, 19, 36). Fourth, cell-mediated immunity, which entails an immune response not involving antibodies, plays a major role in SARS-CoV-2 infection (37). However, this study could not include this aspect during infection risk estimations. Fifth, public health and social measures implemented may affect the infection risk. However, most of measures were relaxed in South Korea during the study period (5).

In summary, our results suggested that hybrid immunity acquired from both SARS-CoV-2 infection and COVID-19 vaccination could provide a higher and more persistent effect humoral immune response against SARS-CoV-2 Omicron infection than infection-induced or vaccine-induced immunity alone. Furthermore, during the predominance period of SARS-CoV-2 Omicron BA.5 and BN.1 variants, hybrid immunity conferred better protection against the SARS-CoV-2 infection compared to that of infection-induced or vaccine-induced immunity alone. Further studies are required to assess longer-term protection against infection by novel SARS-CoV-2 variants using additional COVID-19 booster vaccination.

## Data availability statement

The raw data supporting the conclusions of this article will be made available by the authors, without undue reservation.

## Ethics statement

The studies involving humans were approved by the institutional review boards of Chonnam National University Hospital (IRB No. CNUH-2022-118), Chonbuk National University Hospital (IRB No. CUH-2022-06-031-007), Kyungpook National University Chilgok Hospital (IRB No. KNUCH-2022-05-008-001), and Pusan National University Hospital (IRB No. PNUH-05-2022-103). The studies were conducted in accordance with the local legislation and institutional requirements. Written informed consent for participation in the studies were provided by the participants' legal guardians/next of kin.

## Author contributions

H-WC: Writing – original draft, Writing – review & editing. CA: Formal Analysis, Writing – original draft, Writing – review & editing. JP: Investigation, Writing – review & editing. SL: Investigation, Writing – review & editing. NL: Investigation, Writing – review & editing. C-HJ: Investigation, Writing – review & editing. J-HC: Investigation, Writing – review & editing. HD: Formal Analysis, Writing – review & editing. J-HN: Investigation, Writing – review & editing. J-WL: Project administration, Writing – review & editing. BK: Project administration, Writing – review & editing. SR: Conceptualization, Data curation, Formal Analysis, Methodology, Supervision, Writing – original draft, Writing – review & editing. S-JK: Conceptualization, Data curation, Formal Analysis, Funding acquisition, Investigation, Methodology, Project administration, Resources, Supervision, Writing – original draft, Writing – review & editing.
