# Supplementary material for: Pediatric humoral immune responses and infection risk after severe acute respiratory syndrome coronavirus 2 (SARS-CoV-2) infection and two-dose vaccination during SARS-CoV-2 omicron BA.5 and BN.1 variants predominance in South Korea
Source: Front Immunol. 2023 Dec 20;14:1306604. doi: 10.3389/fimmu.2023.1306604 (PMC10773891; doi:10.3389/fimmu.2023.1306604)
Supplement: Supplementary file 1 [file DataSheet_1.docx]

Supplementary Material


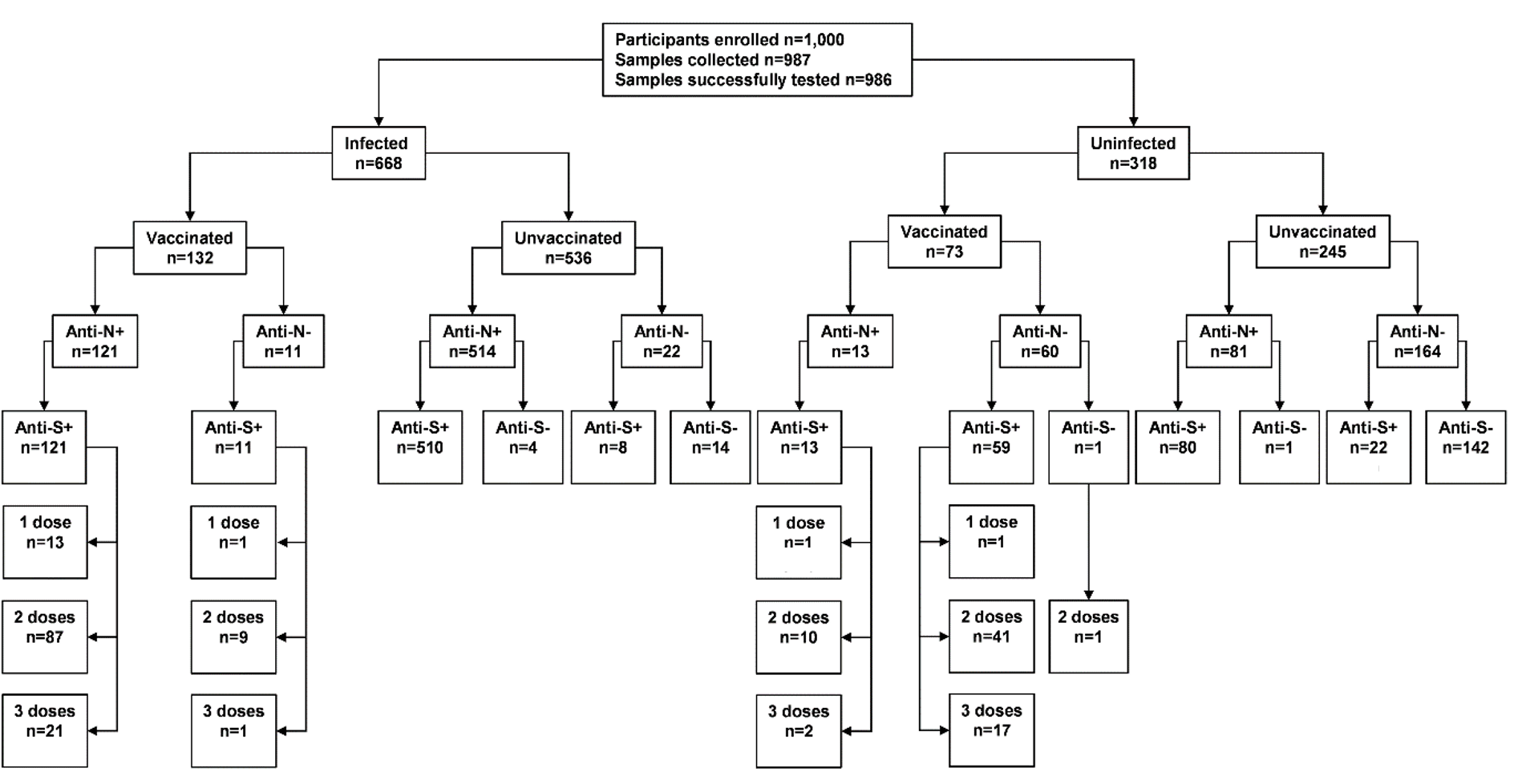
**Supplementary Figure 1.** Study design and flowchart of participants and samples. Letter and number in parenthesis indicate group and number of dose(s), respectively. *Abbreviation*: anti-N-, SARS-CoV-2 nucleocapsid antibody negative; anti-N+, SARS-CoV-2 nucleocapsid antibody positive; anti-S-, SARS-CoV-2 spike antibody negative; anti-S+, SARS-CoV-2 spike antibody positive; n, number of samples.


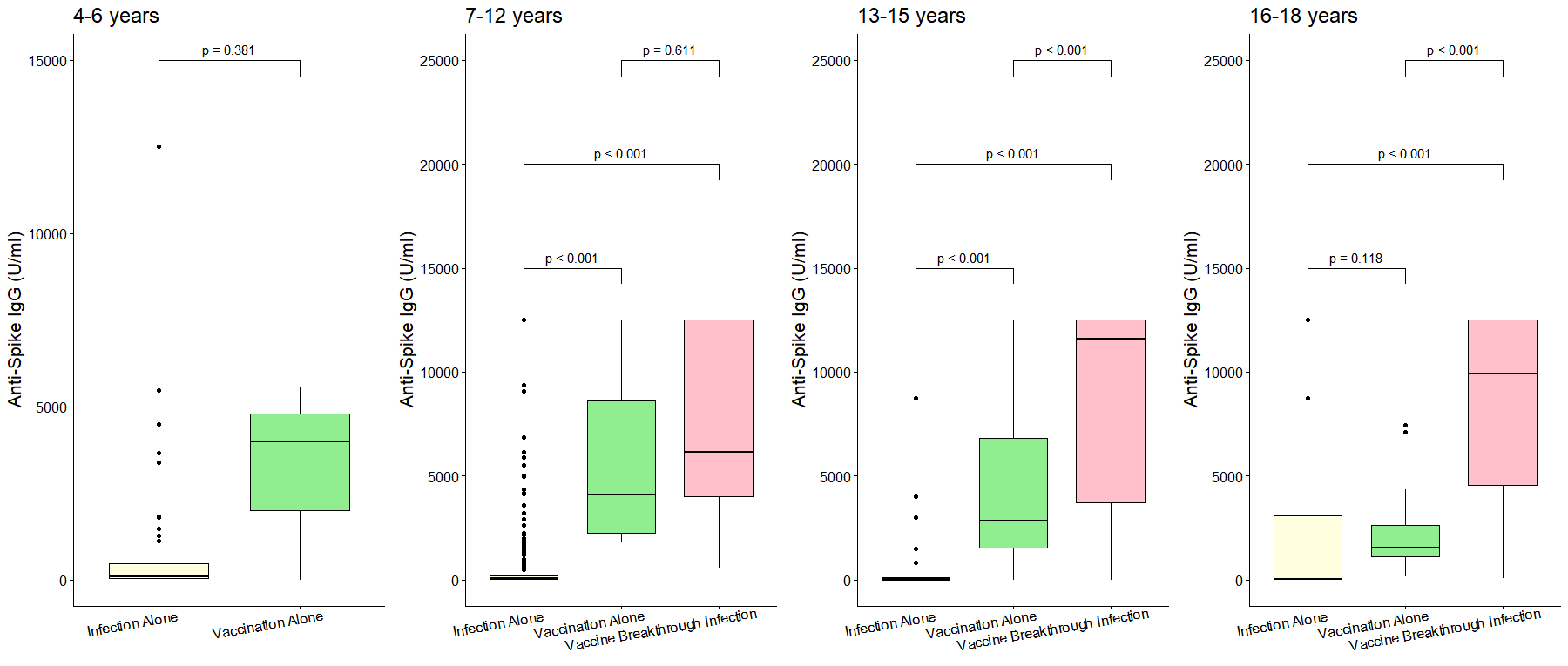


**Supplementary Figure 2.** Unadjusted comparisons of anti-S IgG by category of SARS-CoV-2 infection and/or COVID-19 vaccination in different age groups. *P*-values were determined using the Kruskal-Wallis test. The comparisons do not adjust for sampling time, which is different for every group. The box plots denote the median, the 25th percentile, and the 75th percentile of the anti-S (y-axis) and each group (x-axis) representing SARS-CoV-2 infection alone (light yellow), vaccination alone (light green), and vaccine breakthrough infection (pink).


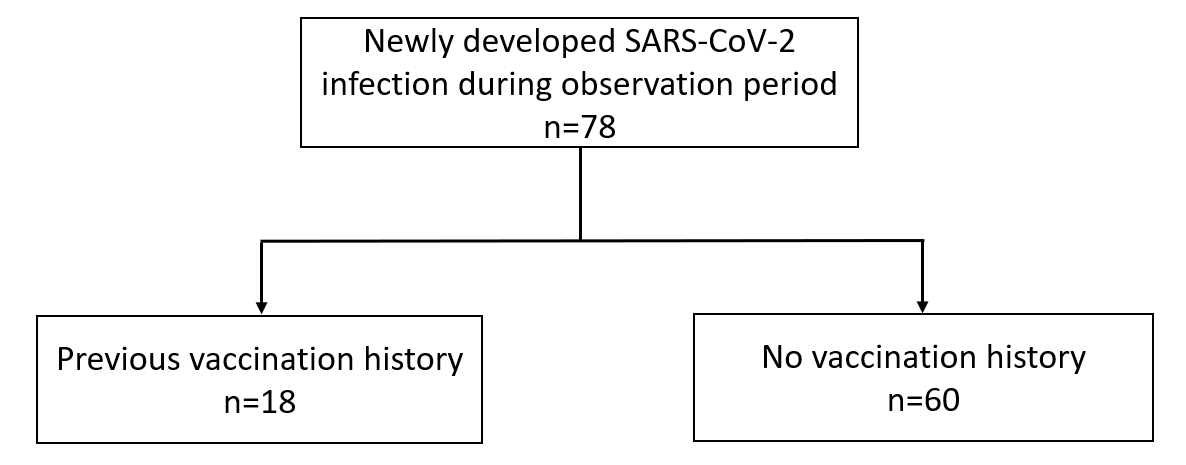


**Supplementary Figure 3.** Flowchart of participants with newly developed SARS-CoV-2 infection.
